# *In vitro* activity of robenidine analogue NCL195 against methicillin-resistant *Staphylococcus pseudintermedius*

**DOI:** 10.1128/spectrum.03187-25

**Published:** 2026-04-30

**Authors:** Sayara Bista, Hang Thi Nguyen, Tatiana P. Soares da Costa, Lucy Woolford, Mark A. T. Blaskovich, Tania Veltman, Kelly A. Young, Adam McCluskey, Meng Siak, Stephen W. Page, Henrietta Venter, Darren J. Trott, Abiodun D. Ogunniyi

**Affiliations:** 1 ARC Training Centre for Environmental and Agricultural Solutions to Antimicrobial Resistance, The University of Queensland 1974, Brisbane, Queensland, Australia; 2 Australian Centre for Antimicrobial Resistance Ecology, School of Animal and Veterinary Sciences, The University of Adelaide 96017, Roseworthy, South Australia, Australia; 3 School of Agriculture, Food and Wine & Waite Research Institute, The University of Adelaide 98479 , Urrbrae, South Australia, Australia; 4 School of Animal and Veterinary Sciences, The University of Adelaide 96017, Roseworthy, South Australia, Australia; 5 Centre for Superbug Solutions, Institute for Molecular Bioscience, The University of Queensland 1974, Brisbane, Queensland, Australia; 6 Chemistry, School of Environmental and Life Sciences, The University of Newcastle 98494, Callaghan, New South Wales, Australia; 7 Perth Animal Dermatology, Western Australia Veterinary Emergency and Speciality, Success, Western Australia, Australia; 8 Neoculi Pty Ltd., Burwood, Victoria, Australia; 9 Health and Biomedical Innovation, Clinical and Health Sciences, University of South Australia 1067 , Adelaide, South Australia, Australia; Seton Hall University, South Orange, New Jersey, USA

**Keywords:** methicillin-resistant *Staphylococcus pseudintermedius*, cell toxicity, robenidine, multidrug resistance, haemolysis, minimal inhibitory concentrations, transmission electron microscopy, antimicrobial stewardship, bactericidal activity, antimicrobial agents

## Abstract

The rise of difficult-to-treat infections among companion animals, particularly the increasing incidence of methicillin-resistant *Staphylococcus pseudintermedius* (MRSP) in dogs poses significant challenges for effective treatment options in veterinary practice. In this study, we demonstrate that the novel robenidine analog NCL195 exhibits antibacterial activity against MRSP isolates collected from dogs, achieving uniform minimal inhibitory concentrations of 1 μg/mL across all 160 isolates from multiple clinical cases and representing diverse sequence types. NCL195 acts by disrupting the bacterial cell membrane potential; no resistant mutants emerged during prolonged serial passage experiments, and transmission electron microscopy showed marked changes to the cell membrane of NCL195-treated MRSP compared to untreated controls. Furthermore, NCL195 showed minimal lysis of dog and sheep red blood cells and moderate toxicity to mammalian cell lines. These results highlight NCL195 as a promising candidate for further development as a novel antimicrobial agent to address the urgent need for effective treatments against MRSP infections in veterinary medicine.

**IMPORTANCE:**

The rise of difficult-to-treat infections among companion animals, particularly the increasing incidence of methicillin-resistant *Staphylococcus pseudintermedius* (MRSP) in dogs, poses significant challenges for effective treatment options in veterinary practice. Here, we show that NCL195, a member of a novel class of pyrimidine compounds, exhibits antibacterial activity against 160 MRSP isolates collected from dogs from multiple clinical cases. Additionally, no resistant mutants developed during prolonged serial passage experiments, and NCL195 showed low lysis of dog and sheep red blood cells. Developing NCL195 as an “animal-only” drug promotes the “responsibility” and “refinement” principles of antimicrobial stewardship.

## INTRODUCTION

A national survey conducted in Australia in 2022 estimated that nearly half of the population owns a dog (1). The rising number of dogs in society may be attributed to evidence indicating that pet ownership can increase physical activity among owners, which in turn is associated with reduced serum cholesterol, lower triglyceride levels, and a decreased incidence of cardiovascular events (2). As dog ownership becomes increasingly common, there is increased risk to human health as dogs can harbor and transmit a variety of pathogens to humans via direct contact, bites, or exposure to contaminated saliva, urine, or feces. This can lead to significant health effects, such as skin infections, gastrointestinal illnesses, and even life-threatening diseases (3). Canine pathogens such as *S. pseudintermedius*, often referred to as the “golden staph of dogs,” can also infect humans and pose significant challenges in both clinical management and public health (4). While this bacterium commonly colonizes the skin and mucosal surfaces of healthy dogs, it is also implicated as the primary causative agent in a range of infections, most notably pyoderma, otitis externa, and urinary tract infections (5).

Recent studies report a sharp rise in antibiotic resistance in *S. pseudintermedius* from dogs (6, 7). These bacteria increasingly withstand antibiotics that are commonly prescribed in dogs, including clindamycin, fluoroquinolones, tetracycline, and trimethoprim-sulfamethoxazole (8). Methicillin-resistant *S. pseudintermedius* (MRSP) isolates are commonly classified as multidrug-resistant (MDR), a term indicating acquired non-susceptibility to at least one agent in three or more antimicrobial categories. Although patterns of antibiotic resistance in MRSP vary geographically, a recent study confirms its status as a widespread MDR pathogen (9). As a consequence, infections caused by MRSP occasionally require the use of critically important human antimicrobials (10). The emergence of multidrug-resistant strains has severely restricted effective treatment options, highlighting the need for alternative therapeutic strategies and stringent implementation of antimicrobial stewardship in veterinary medicine (5).

Beyond the overall rise in resistance, the genetic background of MRSP, specifically its sequence type (ST), plays a crucial role in determining patterns of antimicrobial resistance. A national surveillance in Australia revealed striking clonal diversity among MRSP isolates, with 19 distinct STs identified, and five predominating (ST71, ST497, ST316, ST496, and ST45). Importantly, these major STs are not only geographically clustered but are also associated with multi- to extensively drug-resistant profiles, frequently showing resistance to several antibiotic classes, including fluoroquinolones, tetracyclines, trimethoprim-sulfamethoxazole, and aminoglycosides (6).

The escalating issue of antimicrobial resistance in MRSP urgently necessitates the development of new antibiotics for effective treatment in veterinary medicine. Traditional antimicrobial therapies are increasingly rendered ineffective, leaving clinicians with few alternatives and heightening the risk of untreatable infections. Historically, the compound robenidine (2,2′-bis[(4-chlorophenyl)methylene] carbonimidic dihydrazide hydrochloride) has been used as an anticoccidial agent in poultry and rabbits, with a long record of safety in veterinary settings (11). Although its primary use was not as an antibacterial, its established safety and broad oral administration profile make it an attractive scaffold for repurposing (12). Building on this, a series of novel analogs were synthesized to expand on the antibacterial efficacy of robenidine (13).

The novel entity NCL195, with a 2,4,6-triaminopyrimidine moiety as a bioisosteric replacement for guanidine, exhibits enhanced bactericidal activity against Gram-positive pathogens, including *S. aureus*, *Streptococcus pneumoniae*, and vancomycin-resistant enterococci, acting via disruption of the bacterial cell membrane potential (14). Importantly, NCL195 demonstrated a low propensity for resistance development, limited hemolysis, minimal cytotoxicity to mammalian cells (14), and no observable histological effects in major organs in mouse toxicity trials (15, 16). This positions NCL195 as a promising candidate for combating resistant infections such as those caused by MRSP, where therapeutic options are rapidly diminishing. This study aims to explore NCL195 as a potential treatment for MRSP infections. Developing NCL195 as an “animal-only” drug promotes the “responsibility” and “refinement” principles of antimicrobial stewardship.

## MATERIALS AND METHODS

### Antimicrobial agents and medicinal chemistry

**Fig 1:**
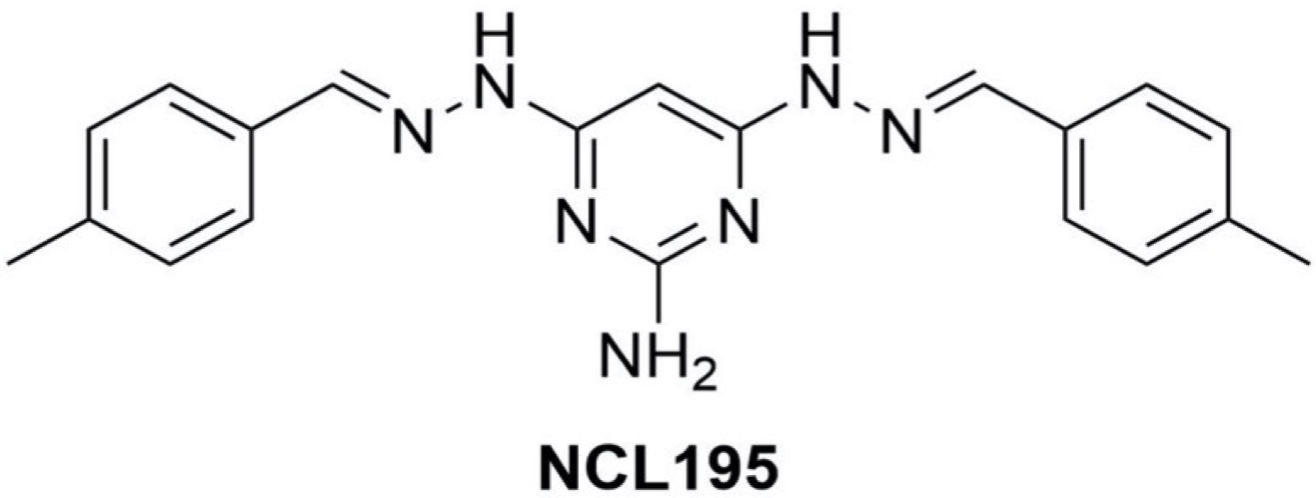
Structure of NCL195 (4,6-bis(2-((E)-4-methylbenzylidene)hydrazineyl)pyrimidine-2-amine).

Robenidine analog NCL195 (Fig. 1) was synthesized in-house at the University of Newcastle as reported previously (14) and stored in a sealed sample container out of direct light at 4°C at the study site at the Clinical and Health Science Laboratory, University of South Australia. Amikacin and vancomycin were purchased from Sigma-Aldrich (Australia). Stock solutions containing 25.6 mg/mL of each compound (amikacin and vancomycin in water, NCL195 in DMSO) were prepared and stored in 1 mL aliquots at −80°C and defrosted immediately prior to use.

### Bacterial strains and growth conditions

The MRSP isolates in this study are listed in Table S1 in Supporting Information. A total of 160 clinical isolates of MRSP were obtained from the Australian Centre for Antimicrobial Resistance Ecology (ACARE) collection at the University of Adelaide. ACARE serves as the national repository for samples submitted by veterinary diagnostic laboratories across Australia, including those from New South Wales, Victoria, South Australia, and Western Australia. This provides a broad geographic source for the collection. All bacteria were stored at −80°C in Luria-Bertani (LB) broth containing 15% (vol/vol) glycerol. Bacterial identities were confirmed by matrix-assisted laser desorption/ionization time-of-flight mass spectroscopy (MALDI-TOF/MS) calibrated with the Bacterial Test Standard *Escherichia coli* (BTS) at ACARE, using a Bruker MALDI Biotyper following the manufacturer’s recommendations prior to experiments. Methicillin resistance was confirmed by oxacillin (1 µg; Oxoid) disc diffusion on Mueller-Hinton agar, using Clinical and Laboratory Standards Institute (CLSI) breakpoints for *S. pseudintermedius*; isolates with inhibition zones ≤18 mm were classified as methicillin-resistant. The isolates were cultured on horse blood agar (HBA) and incubated at 37°C for 18 h.

### Multilocus sequence typing

The multilocus sequence typing (MLST) scheme and the resulting sequence type (ST) assignments for the isolates were performed as previously described by Worthing et al. (17).

### Minimal inhibitory concentration (MIC) determination

The MICs for NCL195, amikacin, and vancomycin on all isolates were determined in duplicate using round-bottom 96-well microtiter plates (Sarstedt 82.1582.001), following the broth microdilution protocol recommended by the Clinical and Laboratory Standards Institute (CLSI), starting with serial twofold dilutions at an initial concentration of 32 μg/mL down to 0.0625 μg/mL (18). Cation-adjusted Mueller-Hinton broth (CAMHB) was used. In addition, serial twofold dilutions of NCL195 were performed in 100% (vol/vol) DMSO, with 1 μL added to each well (1% [vol/vol] DMSO final), as the compound is hydrophobic (13). Duplicate wells containing 1 μL DMSO only were used as growth controls in all experiments. The MICs for vancomycin and amikacin against each isolate were determined for each test to serve as an internal quality control. The MICs of isolates were determined by visual reading and measuring absorbance at *A*_600 nm_ using a Multiskan FC spectrophotometer (Thermo Fisher Scientific). MIC_50_, MIC_90_, and MIC range for NCL195 and the control drugs were also determined for all the MRSP isolates.

### Minimal bactericidal concentration (MBC) determination

The MBCs of NCL195, amikacin, and vancomycin were determined against a subset (40) of the MRSP isolates. Briefly, 10 μL aliquots from each duplicate well of the MIC assays (starting from the MIC for each compound) were inoculated onto an HBA plate and incubated at 37°C. Plates were examined at 24 and 48 h. MBC was recorded as the lowest concentration of each test compound at which a 99.95% colony count (3-log_10_) reduction was observed on the plate (19).

### Time- and concentration-dependent killing assays

A MDR clinical MRSP strain VDL-57 was selected as a representative strain for time- and concentration-dependent kill assay of NCL195 in two biological replicates, following previously described protocols (15) with slight modifications. The isolate was recovered from a canine urine specimen at Adelaide University’s Roseworthy Veterinary Hospital in 2022 and exhibited resistance to multiple drug classes, including 11 non*-β*-lactam (fluoroquinolones [4], aminoglycosides [2], tetracycline [1], sulfonamides [1], amphenicols [1], lincosamides [1], rifamycins [1], and 1 *β*-lactamase inhibitor [amoxiclavulanate]) and 4 *β*-lactam (cefovecin, cefoxitin, ceftriaxone, cephalothin) antibiotics. Few colonies of MRSP VDL-57 from overnight growth on HBA were suspended in saline and adjusted to *A*_600 nm_ of 0.1 (approx. 5 × 10⁷ CFU/mL), then further diluted to a final inoculum of 5 × 10^5^ CFU/mL.

NCL195 was prepared at concentrations equivalent to 1×, 2×, and 4× MIC in 2 mL volumes in a 24-well plate (Sigma-Aldrich, Australia). Amikacin and vancomycin were prepared at 4× MIC to serve as drug controls, and CAMHB only was used as the negative control. Test compounds were initially dissolved in their solvents (NCL195 in DMSO, amikacin, and vancomycin in water) at 100× the target concentration, and 20 μL of the appropriate dilution was added to each 2 mL bacterial preparation. Samples (20 μL) were taken at 0, 0.5, 1, 2, 4, 6, 8, and 24 h, serially diluted tenfold, and plated on HBA. Plates were incubated overnight at 37°C for bacterial count determination (14).

### Point of resistance assay

To assess whether MRSP could develop resistance to NCL195, the assay was performed using two different inocula: MRSP isolate VDL-57 alone, and a mixture of 10 diverse clinical MRSP isolates (details of sequence types and isolation sites are provided in Table S1). The serial passage assay was conducted in 96-well microtiter plates. For each daily passage, MRSP was cultured in wells containing 180 µL of CAMHB with a full series of NCL195 concentrations (0.5×, 0.75×, 1×, 1.5×, 2×, or 4× the MIC). After 24 h of incubation, the plates were visually inspected for bacterial growth. Bacteria from the well corresponding to the highest drug concentration that permitted visible growth were harvested and diluted 1:1,000 in saline. A 20 µL aliquot of this dilution was then used to inoculate a fresh row of wells on a new 96-well plate, each containing the full range of NCL195 concentrations. This daily cycle of identifying the most resistant surviving population and re-challenging it against the full concentration gradient was repeated for 28 days. Amikacin was included as a control at 1×, 4×, and 8× the MIC, with each condition set up in quadruplicate (20). After the 28th passage, cultures were centrifuged at 4,000 × *g* for 10 min and washed twice with 50 mL phosphate-buffered saline (PBS) to eliminate residual antibiotic, bacterial metabolites, and media components. The washed cells were then resuspended in PBS to *A*_600 nm_ of 0.1, and MIC assays were carried out as previously described.

### Transmission electron microscopy (TEM) to view the effect of NCL195 against MRSP

MRSP VDL-57 was treated with 1 μg/mL, 2 μg/mL, or 4 μg/mL of NCL195 for 1 h, and morphological appearance of the cell membrane was determined using TEM, with a slight modification of Procedure 2 described previously (21). Briefly, the cells were washed with PBS + 4% sucrose, fixed in 4.0% formaldehyde, 1.25% glutaraldehyde, 0.01 M CaCl_2_, 4% sucrose, 0.035% ruthenium red, and 0.075% L-lysine acetate, followed by post-fixation in 1% osmium tetroxide; 0.035% ruthenium red in Epon-Araldite. Sections were cut to 1 μm using a glass knife, stained with 1% toluidine blue containing 1% borax, and viewed under a light microscope at 400× to identify the presence of stained bacteria. Thereafter, uranyl acetate- and Reynolds lead citrate-stained ultrathin sections were then viewed on a Tecnai G2 Spirit (FEI Company, Hillsboro, OR, USA) transmission electron microscope operated at 100 kV at Adelaide Microscopy, the University of Adelaide. A full description of the procedure is provided in the Supplementary methods.

### Mechanism of action studies

To investigate how NCL195 affects the membrane integrity of MRSP, changes in membrane potential of four isolates (VDL-57, VDL-76, VDL-79, and VDL-97) were assessed using the fluorescent dye 3,3′-dihexyloxacarbocyanine iodide (DiOC₂(3)) as described previously (22, 23). Briefly, the isolates from overnight culture on HBA were sub-cultured into fresh CAMHB and incubated at 37°C until *A*_600 nm_ reached 0.5. Cells were harvested by centrifugation at 2,900 × *g* for 10 min at 4°C, washed twice with 50 mM potassium phosphate buffer (pH 7.0), and resuspended in the same buffer to a final *A*₆₀₀ _nm_ of 6. For the fluorescence assay, 0.2 mL of each bacterial suspension was added to 1.8 mL of the same buffer in a quartz cuvette (Hellma fluorescence cuvettes, Z600172, Sigma-Aldrich, Australia), and the mixture was stirred gently for 15 min in the presence or absence of 16 µg/mL of the NCL195, representing 16× MIC against MRSP; ampicillin and vancomycin at the same concentration served as controls. The cuvette was then placed into a PerkinElmer LS 55 Fluorescence Spectrometer, with the excitation wavelength set at 488 nm and emission monitored at 620 nm (slit widths: 5 nm for excitation and 7 nm for emission). After measuring baseline fluorescence for 1 min, DiOC₂ (3) was added to a final concentration of 10 µM, and fluorescence was tracked until it plateaued. Cells were subsequently energized with 0.5% (vol/vol) glucose, and changes in fluorescence were recorded until another plateau was reached. Finally, 10 µM of the protonophore carbonyl cyanide *m*-chlorophenyl hydrazone (CCCP) was added to disrupt the membrane potential, and fluorescence was monitored once more until it plateaued.

### Hemolysis assay

Hemolytic activity of NCL195 was performed using fresh dog and sheep red blood cells (RBCs) obtained from Roseworthy Veterinary Hospital, the University of Adelaide, essentially as described previously (14). Fresh RBCs were washed in PBS three times at 500 × *g* for 5 min and then resuspended at 1% (wt/vol) in PBS. Two microliters of serial twofold dilutions of each compound were added into the respective wells, in quadruplicates, in a round-bottom 96-well microtiter tray (Sarstedt 82.1582.001), starting at 128 μg/mL for NCL195 using amikacin and ampicillin as controls. Thereafter, 198 μL of the 1% (vol/vol) RBC solution was added into each well, and the mixture was incubated for 1 h at 37°C, with shaking at 100 rpm. Quadruplicate wells containing either 1% (vol/vol) Triton X-100 or PBS only served as controls. After incubation, the trays were centrifuged at 1,000 × *g* for 3 min, and 100 μL of supernatant from each well was transferred into a new 96-well tray. Absorbance was measured at *A*_450 nm_ using a Multiskan FC Spectrophotometer (Thermo Fisher Scientific) and plotted against each dilution. Hemolytic titer was determined as the reciprocal of the dilution at which 50% of erythrocytes were lysed at *A*_450 nm_ (14). The experiment was performed on two independent occasions.

### *In vitro* cytotoxicity assays

To evaluate the potential cytotoxicity of NCL195 toward mammalian cells, we employed an *in vitro* cytotoxicity assay based on the methodology described previously (14). In this previous study, a range of established adherent mammalian cell lines, specifically Caco-2 (human colorectal adenocarcinoma), HEL299 (non-cancerous human lung fibroblast), Hep G2 (human hepatocellular carcinoma), MDBK (normal bovine kidney), and MCF-7 (human mammary adenocarcinoma), were utilized to assess off-target effects. In this study, MDCK (Madin-Darby canine kidney), Vero (African green monkey kidney), and HFF-1 (human foreskin fibroblasts) cell lines were used to further investigate the safety profile of NCL195.

MDCK, Vero, and HFF-1 cell lines were cultured in Dulbecco’s Modified Eagle’s Medium (DMEM) supplemented with 10% (vol/vol) fetal bovine serum (FBS) and 1% (vol/vol) PenStrep (100 U/mL penicillin and 100 μg/mL streptomycin), maintained at 37°C in 5% CO₂, and sub-cultured every 2–3 days. For cytotoxicity assays, cells were seeded in flat-bottom 96-well tissue culture trays (Sarstedt 83.3924) at densities ranging from 2.5 × 10⁴ to 5 × 10⁴ cells per well. After 24 h of initial incubation, the medium was replaced with fresh DMEM containing 10% (vol/vol) FBS, and cells were allowed to equilibrate for 2 h before treatment. Serial dilutions of NCL195, prepared in DMSO, were added to designated wells (final DMSO concentration ≤1% [vol/vol]), with parallel wells containing DMSO alone serving as negative controls and ampicillin as positive controls. The plates were then incubated for a further 24 h. Subsequently, cell viability was quantified using the WST-1 assay, by adding WST-1 reagent to each well (final concentration 10% [vol/vol]) and measuring absorbance at 450 nm after 1 h. Dose-response curves were generated, and the IC_50_ value for NCL195 and cell lines was determined using non-linear regression analysis (GraphPad Prism v6 software) (14).

## RESULTS

The present study extends previous investigations on the broad-spectrum antibacterial activity of NCL195 to include MRSP, a significant canine pathogen exhibiting increasing multidrug resistance and limited treatment options.

### NCL195 exhibits antibacterial activity against MRSP

The activity of NCL195 against 160 clinical isolates of MRSP from multiple clinical cases and diverse sequence types showed that its MIC was 1 μg/mL for all the 160 isolates, with no isolates exhibiting MIC values below or higher than this concentration (Table 1; Table S1). MBC was also carried out against 40 of the isolates, and the MBC range was found to be 1–2 μg/mL for NCL195 (Table 1; Table S1). Additionally, amikacin and vancomycin (used as control drugs) showed MIC ranges of 1–8 μg/mL and 0.5–1 μg/mL, respectively, and MIC_90_ values of 4 μg/mL and 1 μg/mL, respectively (Table 1).

**TABLE 1:** MIC range values and MIC_90_ for NCL195, amikacin, and vancomycin against MRSP

Compounds	Range (µg/mL)		MIC_90_ (µg/mL) *n* = 160
	MIC (*n* = 160)	MBC (*n* = 40)	
NCL195	1	1–2	1
Vancomycin	0.5–1	0.5–2	1
Amikacin	1–8	1–8	4

### NCL195 kills MRSP without detectable resistance

**Fig 2:**
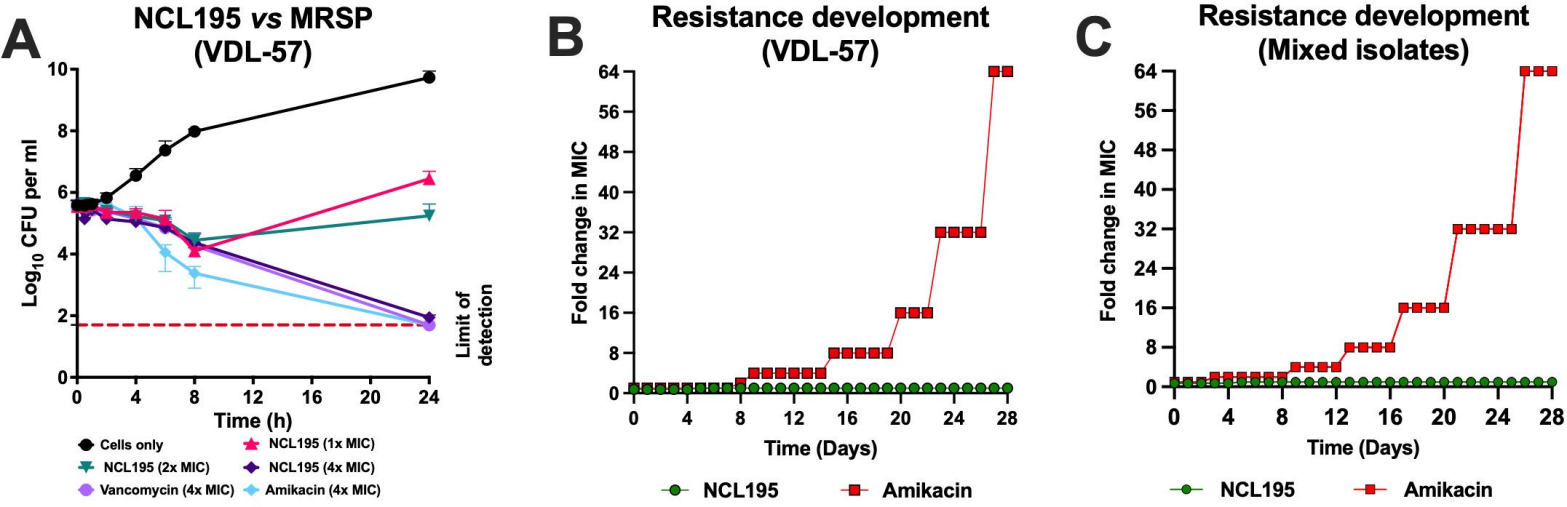
Time-kill kinetics and point of resistance assays. (**A**) NCL195 was prepared at 1×, 2×, and 4× MIC (using 4× MIC of amikacin and vancomycin as controls) in CAMH broth for MRSP VDL-57. Cultures were incubated at 37°C with agitation at 200 rpm. Samples were withdrawn at indicated times and plated on HBA overnight at 37°C with aeration for bacterial enumeration. (**B** and **C**) MRSP VDL-57 (**B**) and mixed MRSP isolates (**C**) were grown in CAMH broth in the presence of 0.5× MIC, 0.75× MIC, 1× MIC, 1.5× MIC, 2× MIC, and 4× MIC of NCL195, using 1× MIC, 2× MIC, 4× MIC, and 8× MIC of amikacin as a control.

We interrogated the time-kill profile of NCL195 against MRSP isolate VDL-57 using 1×, 2×, and 4× MIC of NCL195, with 4× MIC of amikacin and 4× MIC of vancomycin as controls. We found that it took 24 h to reduce the test population of MRSP VDL-57 below the limit of detection at 2× and 4× MIC of NCL195, which was comparable to that of amikacin and vancomycin, as both the control drugs required 24 h to kill bacteria at 4× MIC (Fig. 2A).

The potential for MRSP to develop resistance to NCL195 was investigated over 28 consecutive daily passages for both the VDL-57 isolate and the mixture of 10 clinical isolates. For NCL195, bacterial growth was consistently observed at sub-inhibitory concentrations (0.5× to 0.75× MIC). However, even after 28 days of continuous exposure, the bacteria were unable to grow at concentrations above 0.75× MIC. MIC testing of the cultures from the final passage confirmed that there was no change from the baseline MIC, indicating that no resistant mutants had been selected. In contrast, resistance to amikacin increased, reaching 4× MIC by day 9, 8× MIC by day 15, 16× MIC by day 20, 32× MIC by day 23, and 64× MIC by day 27 for VDL-57, and reaching 4× MIC by day 8, 8× MIC by day 12, 16× MIC by day 16, 32× MIC by day 20, and 64× MIC by day 24 for the mixed isolates (Fig. 2C). The absence or presence of resistance was verified through MIC testing of bacteria cultured at each concentration.

### NCL195 exerts its antibacterial action on the cell membrane of MRSP

**Fig 3:**
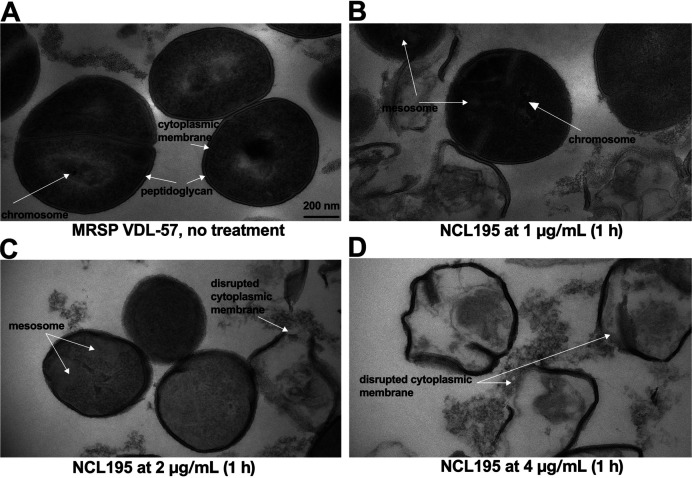
Effect of NCL195 on the morphology of MRSP VDL-57. TEM showing visual differences between the cell membranes of untreated (**A**) and treated (**B–D**) MRSP VDL-57 samples. The cell membranes of VDL-57 cells were exposed to (**B**) 1 μg/mL, (**C**) 2 μg/mL, and (**D**) 4 μg/mL NCL195 for 1 h before processing for TEM.

TEM was utilized to investigate NCL195’s antibacterial mechanism against MRSP VDL-57 following 1 h exposure at 1, 2, and 4 μg/mL concentrations. Untreated cells displayed typical Gram-positive ultrastructure (Fig. 3A). However, NCL195 induced dose-dependent ultrastructural damage at 1 μg/mL; the initial disruption included prominent mesosome-like structures (cytoplasmic membrane invaginations) and chromosomal disorganization (Fig. 3B). Increasing the dose to 2 μg/mL intensified mesosome-like structure formation and cytoplasmic electron density alterations, indicative of cellular stress (Fig. 3C). At 4 μg/mL, severe damage was observed, characterized by extensive cytoplasmic membrane and peptidoglycan disruption, significant cytoplasmic leakage, and a severely compromised cellular architecture (Fig. 3D). These findings strongly suggest that NCL195 targets and progressively disrupts the bacterial cytoplasmic membrane, leading to lethal structural damage.

### NCL195 disrupts the membrane potential of MRSP

**Fig 4:**
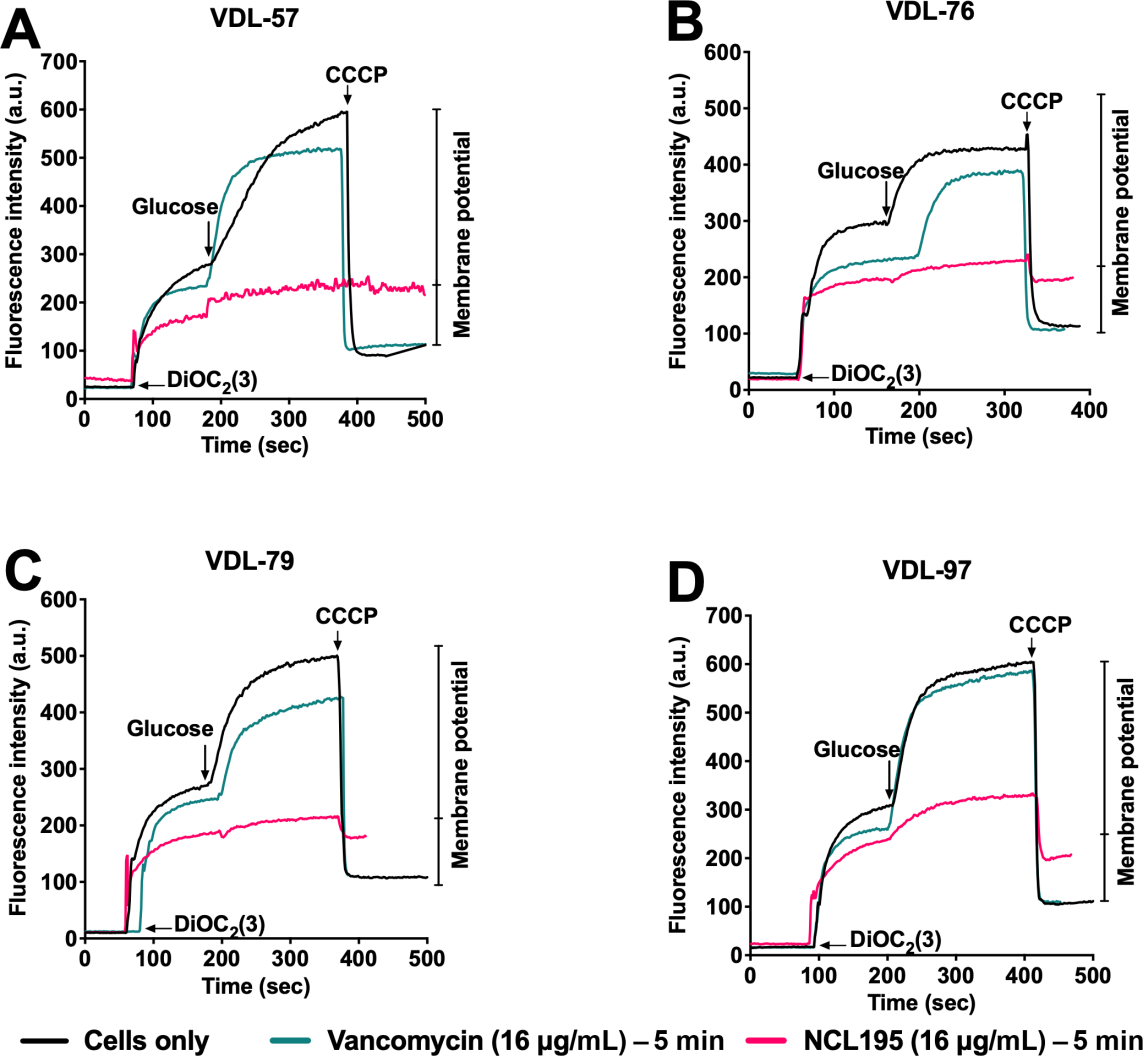
Dissipation of the membrane potential of four MRSP isolates by NCL195: (**A**) VDL-57, (**B**) VDL-76, (**C**) VDL-79, and (**D**) VDL-97. Bacterial suspensions were exposed to 16 μg/mL NCL195 and vancomycin (control) for 5 min, after which DiOC_2_(3) was added and the fluorescence monitored until it plateaued. Cells were then re-energized with 0.5% (vol/vol) glucose, and the establishment of membrane potential was measured as an increase in fluorescence until it plateaued. The membrane potential was then disrupted by the addition of the proton ionophore (CCCP).

To further investigate whether NCL195 perturbs the bacterial cell membrane, we assessed the ability of MRSP cells to establish and maintain a membrane potential (Δψ) in the presence of these compounds using the fluorescent membrane potential probe 3,3-diethyloxacarbocyanine iodide (DiOC₂(3)). When MRSP VDL-57, VDL-76, VDL-79, and VDL-97 were challenged with 16 µg/mL of NCL195 for 5 min, there was a significant reduction in membrane potential, confirming its membrane-disruptive activity in MRSP compared to that of the untreated cells and cells in the presence of vancomycin (Fig. 4).

### NCL195’s hemolytic activity and cytotoxicity to mammalian cell lines

**Fig 5:**
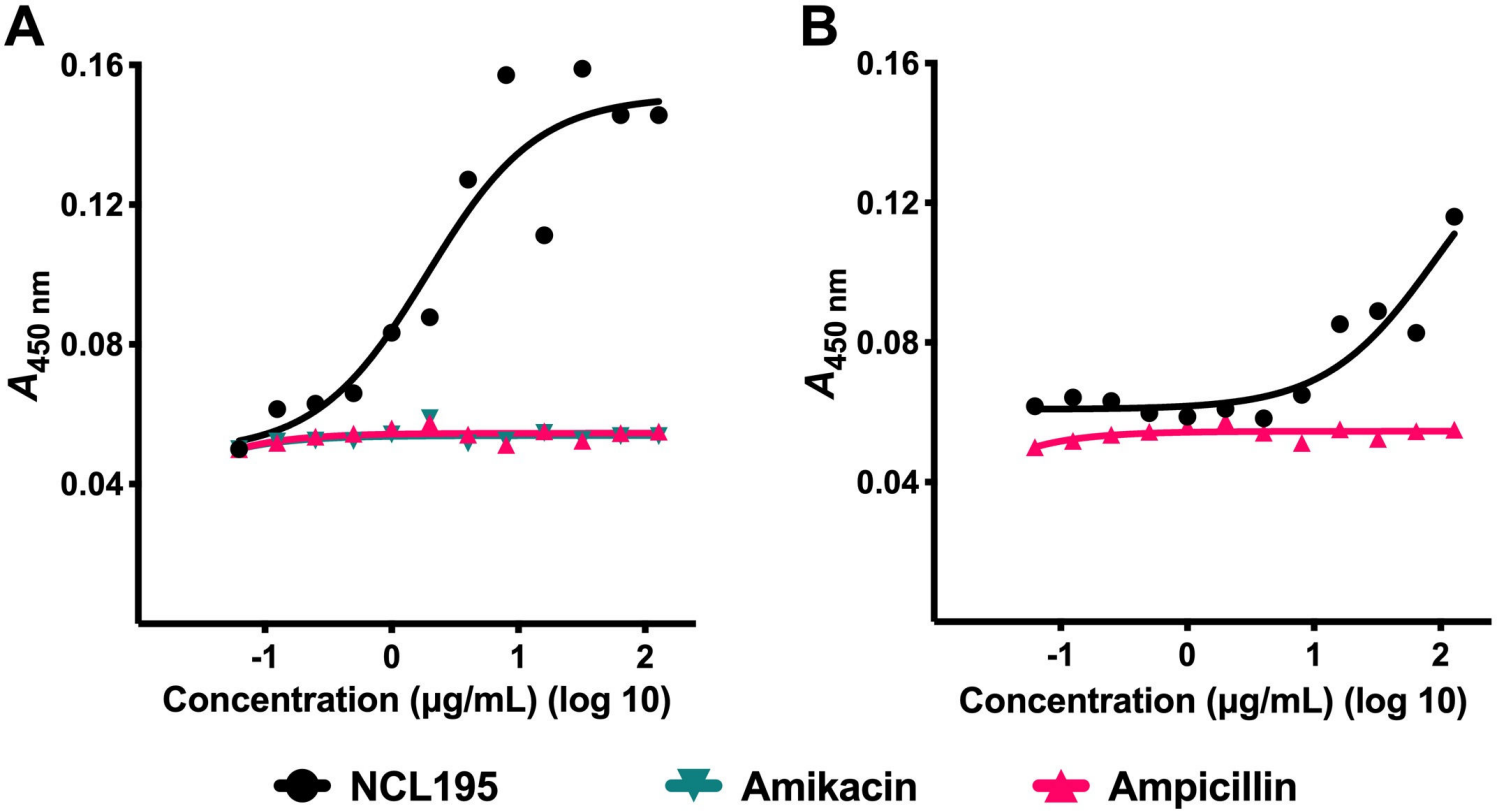
NCL195 demonstrates moderate toxicity to dog and sheep RBCs. Hemolysis of dog RBCs (**A**) and sheep RBCs (**B**). Dog and sheep RBCs were treated with NCL195 and control drugs (amikacin and ampicillin) up to the highest concentration of 128 μg/mL for 2 h at 37°C with shaking at 100 rpm. 1% (vol/vol) Triton X-100 or PBS only served as controls. After incubation, the trays were centrifuged at 1,000 × *g* for 3 min, and 100 μL of supernatant from each well was transferred into a new 96-well tray, and absorbance was measured at *A*_450 nm_. The absorbance value for Triton X-100 = 1.0, indicating complete RBC lysis. The absorbance values for NCL195 at the highest concentration were ≈ 0.16–0.18, corresponding to <20% hemolysis, whereas amikacin and ampicillin caused negligible lysis.

The hemolytic activity of NCL195 against dog and sheep RBCs was evaluated. For both dog and sheep RBCs, the 50% hemolytic titer HC_50_ was greater than 128 μg/mL (the highest concentration tested). These values suggest that NCL195 is generally well tolerated by RBCs. Notably, low-level hemolysis HC_10_ for dog RBCs was observed at 10 μg/mL, indicating an early onset of hemolysis. However, even at a high concentration (128 μg/mL), there was less than 20% lysis (Fig. 5).

The toxicity profile of NCL195 in Caco-2, HEL299, Hep G2, MDBK, and MCF-7 cell lines has been published previously (14). Therefore, only the cytotoxicity findings for MDCK, Vero, and HFF-1 cell lines are presented here. The results of the *in vitro* cytotoxicity assays demonstrated that the IC_50_ values of NCL195 against all three tested cell lines were between 8 and 16 μg/mL, indicating moderate toxicity (Table 2).

**TABLE 2:** IC_50_ data for NCL195 against MDCK, Vero, and HFF-1 cell lines

Cell lines	IC_50_ (μg/mL)	
	NCL195	Ampicillin
MDCK	16	>64
Vero	16	>64
HFF-1	8	>64

## DISCUSSION

MRSP is a significant bacterial pathogen in dogs and is becoming increasingly difficult to treat due to its resistance to multiple antibiotics. The rising prevalence of these drug-resistant strains severely limits treatment options and can lead to serious infections that are difficult to control. Addressing this ongoing problem, we investigated the potential of the robenidine analog NCL195 as an antimicrobial agent against a wide range of clinical MRSPs, building on the known safety profile of its parent compound and introducing structural modifications specifically incorporating a 2-amino-4,6-dihydrazinopyrimidine moiety to enhance both potency and drug-like characteristics (14). These findings extend previous work demonstrating potent activity of NCL195 against *S. aureus* and *Streptococcus pneumoniae* (14) to *S. pseudintermedius*, indicating that this compound maintains broad activity against multiple Gram-positive pathogens.

Our current study shows that NCL195 is highly effective against a large and diverse set of MRSP isolates from dogs, demonstrating a remarkably consistent MIC of just 1 μg/mL across all the 160 clinical isolates tested, and MBC values of 1–2 μg/mL for a subset (40) of the isolates. We have confirmed these results in duplicate assays and verified that the MICs for amikacin and vancomycin fell within expected ranges, supporting the robustness of the assay. This is particularly notable given that previous studies reported MIC values for NCL195 ranging from 1 to 8 μg/mL for other Gram-positive bacteria such as *S. pneumoniae* and *S. aureus* (14). The lower and more uniform MIC values against MRSP suggest that NCL195 may be even more potent against this important veterinary pathogen. When compared to standard treatment options, NCL195 performed as well as vancomycin and was more consistent and effective than amikacin, which showed a broader MIC range and a higher MIC_90_. Recent research into benzguinols A and B, which are semisynthetic derivatives of a fungal natural product, revealed similarly low MICs (0.5–1 μg/mL) against MRSP, reflecting strong antibacterial potential, but their MBCs were considerably higher (4–8 μg/mL), indicating that higher concentrations were required to kill the bacteria (24). In contrast, NCL195’s ability to both inhibit and effectively kill MRSP at the same low concentration highlights its antibacterial effect.

Our study demonstrates that NCL195 exhibits antibacterial activity against MRSP, reducing bacterial numbers below the limit of detection within 24 h at 2× MIC and 4× MIC comparable to what we observed with the commonly used antibiotics amikacin and vancomycin at 4× MIC. Notably, this time-kill kinetics in MRSP is broadly consistent with previous findings for *S. aureus* and *S. pneumoniae*, where NCL195 was also able to achieve rapid and complete bacterial clearance at the respective effective concentrations (14). This suggests that NCL195 can reliably kill MRSP within 24 h at effective concentrations. Beyond its antibacterial effect, NCL195 exhibited notable stability with respect to resistance development. In our prolonged exposure experiments, which involved 28 consecutive daily passages of mixed MRSP isolates under a range of sub-inhibitory and inhibitory concentrations, no resistant mutants emerged at concentrations above 1× MIC. This stability contrasts sharply with the increasing resistance observed for amikacin under similar conditions, where the MIC rose substantially over the same period. Resistance mechanisms to amikacin, due to the acquisition of aminoglycoside-modifying enzyme genes such as *aph (3)-IIIa*, have been described previously (25). In contrast, we did not see any resistance to NCL195 in our study, likely because the bacterial population lacks known mechanisms to resist this compound, or alternatively, because NCL195 has multiple targets on the bacterial membrane, or perhaps because mutations leading to resistance are lethal. The finding that *S. pseudintermedius* did not develop resistance to NCL195, even under prolonged selective pressure, suggests that it could be an effective and long-lasting therapeutic option against multidrug-resistant infections.

Our TEM results on MRSP VDL-57 demonstrate that NCL195 induces dose-dependent ultrastructural damage, characterized by initial mesosome formation, progressing to severe cytoplasmic membrane disruption and leakage. This membrane-targeting mechanism aligns with previous studies by references (15) and (14), further confirming that NCL195 perturbs the bacterial cell membrane. Our cell membrane potential studies confirm that NCL195 acts against MRSP by disrupting its cytoplasmic membrane potential. Fluorescent membrane potential assays revealed a significant decrease in membrane potential in MRSP cells treated with NCL195 compared to untreated and vancomycin-treated controls, suggesting loss of membrane function and disruption of essential cellular processes. This observation is consistent with previous studies in *S. pneumoniae* and *S. aureus*, where NCL195 permeabilizes the cytoplasmic membrane (14). The demonstration of this membrane-disrupting mechanism in MRSP not only aligns with the proposed mode of action in other Gram-positive pathogens but also suggests that the efficacy of NCL195 results from its action on a vital cellular structure. Overall, these results strengthen the case for NCL195 as a promising antibacterial agent against Gram-positive bacteria, with a membrane-active mechanism of action.

NCL195 demonstrated a moderate cytotoxicity profile in both red blood cell and mammalian cell assays. In hemolysis experiments, it showed moderate toxicity to dog and sheep RBCs, with less than 50% lysis even at concentrations exceeding 128 μg/mL. This low hemolytic activity was comparable to that of amikacin and ampicillin, suggesting that NCL195 is generally well tolerated by erythrocytes. Consistent with these findings, previous studies using human RBCs also reported an HC_50_ value greater than 128 μg/mL for NCL195 (14). *In vitro* cytotoxicity assays indicated a selectivity index in the range of 8–16 g/mL, consistent with a moderate therapeutic window for systemic use. Further medicinal chemistry optimization will be required to improve the selectivity index before systemic administration can be considered. Additionally, it has been argued by others that *in vitro* cytotoxicity is not necessarily predictive of *in vivo* toxicity and that the true picture of NCL195’s toxicity can only be determined by *in vivo* toxicity testing (26–28). Despite the potential cell toxicity observed, NCL195 was previously tested in systemic infection models, where it showed efficacy without toxicity (15, 16). Given that *S. pseudintermedius* is primarily a canine pathogen, future *in vivo* studies in dogs will be essential to evaluate the safety, pharmacokinetics, and therapeutic efficacy of NCL195.

NCL195 has been shown previously to possess high metabolic stability and favorable pharmacokinetic properties, including low degradation in human and mouse liver microsomes, high plasma concentrations, prolonged elimination half-life, and low plasma clearance rates (14). Combined with its strong *in vitro* efficacy and moderate toxicity, these features highlight NCL195’s potential as a therapeutic antimicrobial agent against MRSP. Building on these promising properties and efficacy, the next steps include *in vivo* efficacy and safety studies in animal models to support the advancement of NCL195 toward veterinary clinical use.

### Conclusions

The findings of this study highlight the promising therapeutic potential of NCL195 as a novel antimicrobial agent against MRSP. NCL195 exhibited potent and consistent antibacterial activity across a broad collection of clinical MRSP isolates, with low minimal inhibitory concentrations. Its unique membrane-disrupting mechanism, moderate cytotoxicity to mammalian cells, and lack of resistance development position NCL195 as a strong candidate for further development in veterinary medicine. These results support continued preclinical and *in vivo* investigations to advance NCL195 toward clinical use for combating multidrug-resistant infections in companion animals.

## Data Availability

All the data generated in this manuscript are in the main text and supplementary information.

## SUPPLEMENTAL MATERIAL

Supplemental material spectrum.03187-25-s0001.docx Supplemental methods; Table S1.

ASM does not own the copyrights to Supplemental Material that may be linked to, or accessed through, an article. The authors have granted ASM a non-exclusive, world-wide license to publish the Supplemental Material files. Please contact the corresponding author directly for reuse.
