# Supplementary material for: In vitro activity of robenidine analogue NCL195 against methicillin-resistant Staphylococcus pseudintermedius
Source: Microbiol Spectr. 2026 Apr 30;14(6):e03187-25. doi: 10.1128/spectrum.03187-25 (PMC13228035; doi:10.1128/spectrum.03187-25)
Supplement: Supplemental material — Supplemental methods; Table S1. [file spectrum.03187-25-s0001.docx]

**Supporting Information**

**Supplementary Methods**

*Preparation of bacterial cells.* MRSP-VDL57 was sub-cultured twice in HBA. Several colonies from the overnight-cultured HBA were grown in fresh CAMH broth and then incubated at 37 °C under continuous agitation until an *A*_600 nm_ reading of 0.5 was reached. The bacteria were then diluted (5 × 10^7^ CFU/mL) in working concentrations of 1 μg/mL, 2 μg/mL or 4 μg/mL of NCL195 for 1 h.

*Sample Processing for TEM.* The TEM protocol was modified (Procedure 2) from a previous study (1). Briefly, all samples were centrifuged at 2,900 × g at 4 °C for 10 min and washed twice using PBS supplemented with 4% sucrose, then immediately fixed using fixatives (4.0% paraformaldehyde, 1.25% glutaraldehyde, 0.01M CaCl_2_ and 4% sucrose, 0.035% ruthenium red and 0.075% L-lysine acetate, followed by post-fixation in 1% osmium tetroxide and 0.035% ruthenium red in Epon-Araldite for 1 h at room temperature. Subsequently, all samples were washed twice in cacodylate buffer. Then, cells were dehydrated using a graded series of 50%, 70%, 90% and 100% ethanol for 10 min (2 × for each step and 3 × for 15 min in 100% ethanol) at room temperature. Thereafter, the cells were infiltrated with propylene oxide and Epon-Araldite resin (50:50, v/v). Samples were incubated in 100% Epon-Araldite resin overnight at room temperature under rotation. Subsequently, the cells were polymerized in fresh Epon-Araldite resin at 70 °C for 48 h. Sections were cut to 1 μm using a glass knife, stained with 1% toluidine blue containing 1% borax and viewed under a light microscope at 400 × magnification to identify stained bacteria. At least four ultra-thin sections were then cut to 80 nm with an ultramicrotome (Leica) using a diamond knife (Diatome) and placed on 200-mesh copper EM grids (Proscitech). Sections were sequentially stained with uranyl acetate (4% in dH_2_O), and Reynolds leads citrate for 10 min each, with three washes in distilled water in between each stain. Sections were then viewed between 25000 × and 130000 × on a Tecnai G2 Spirit 120 kV Transmission Electron Microscope (FEI Company). Images were obtained at 30000× magnification and analyzed using Olympus Soft Imaging Systems at Adelaide Microscopy, The University of Adelaide.

**Supplementary Results**

**Table S1**. MRSP isolates, sources, Matrix-assisted laser desorption/ionisation time of-flight mass spectroscopy (MALDI-TOF/MS) results, MIC values, MBC values, MIC range, MIC_50_ and MIC_90_ (μg/ml) of NCL195, Vancomycin and Amikacin

| **Isolates**  **(VDL no)** | **Ref no.** | **Methicillin** | **MALDI-TOF/MS** | **Site of Isolation** | **MLST** | **MIC (MBC) (μg/ml)** | | |
| --- | --- | --- | --- | --- | --- | --- | --- | --- |
|  |  |  |  |  |  | **NCL195** | **Van** | **Ami** |
| 1 | 23-00298 | R | P | skin | ND | 1 | 0.5 | 8 |
| 2 | 23-00800 | R | P | skin | ND | 1(1) | 0.5(1) | 8(8) |
| 3 | 23-00853 | R | P | surgical site implant | ND | 1 | 1 | 8 |
| 4 | 23-07042 | R | P | ear | ND | 1(1) | 0.5(0.5) | 8(8) |
| 5 | 23-07052 | R | P | pyoderma | ND | 1 | 0.5 | 8 |
| 6 | 23-07121 | R | P | skin | ND | 1(1) | 0.5(0.5) | 8(8) |
| 7 | 23-07136 | R | P | urine | ND | 1 | 0.5 | 8 |
| 8 | 23-07163 | R | P | pyoderma | ND | 1(1) | 1(1) | 8(8) |
| 9 | 24-01599 | R | P | implant | ND | 1 | 0.5 | 8 |
| 10 | 24-01803 | R | P | feeding tube site | ND | 1(1) | 1 (1) | 8(16) |
| 11 | 24-01887 | R | P | paw tissue | ND | 1 | 0.5 | 8 |
| 12 | 24-02129 | R | P | lip dermatitis | ND | 1(1) | 0.5 | 8(8) |
| 13 | 24-02306 | R | P | paw and nail swab | ND | 1 | 0.5 | 8 |
| 14 | 24-02504 | R | P | skin | ND | 1(1) | 0.5(0.5) | 8(8) |
| 15 | 24-02503 | R | P | urine calculi | ND | 1 | 1 | 8 |
| 16 | 24-02724 | R | P | vulval swab | ND | 1(1) | 1(2) | 8(16) |
| 17 | 24-03047 | R | P | ear | ND | 1 | 1 | 8 |
| 18 | 24-03194 | R | P | tissue | ND | 1(1) | 1(2) | 8(8) |
| 19 | 24-03314 | R | P | nasal | ND | 1 | 0.5 | 8 |
| 20 | 24-03318 | R | P | wound swab | ND | 1(1) | 0.5(0.5) | 8(16) |
| 21 | ex IDEXX | R | P | dog joint | ND | 1(1) | 0.5(2) | 2(4) |
| 22 | 17-1962 | R | P | MRSP ex IDEXX Seaside vets | ND | 1 | 1 | 4 |
| 23 | 17-2399 | R | P | MRSP | ND | 1 | 1 | 4 |
| 24 | 18-2572 | R | P | ear | ND | 1(1) | 1(1) | 4(4) |
| 25 | 18-2573 | R | P | ear | ND | 1 | 1 | 4 |
| 26 | 19-0060 | R | P | skin | ND | 1(1) | 0.5(1) | 4(8) |
| 27 | 19-0336 | R | P | skin | ND | 1 | 0.5 | 4 |
| 28 | 19-00989 | R | P | MRSP ex Gribbles | ND | 1 | 0.5 | 2 |
| 29 | 19-01200 | R | P | skin swab | ND | 1 | 0.5 | 4 |
| 30 | 19-1813 | R | P | furuncle swab | ND | 1(1) | 1(1) | 4(8) |
| 31 | 19-2501 | R | P | abscess | ND | 1 | 1 | 4 |
| 32 | 20-0232 | R | P | MRSP CAHC Peter Hill | ND | 1 | 0.5 | 2 |
| 33 | 20-0231 | R | P | MRSP | ND | 1 | 0.5 | 4 |
| 34 | 20-0273 | R | P | MRSP | ND | 1 | 0.5 | 4 |
| 35 | 20-01600 | R | P | inguinal area | ND | 1 | 0.5 | 2 |
| 36 | 20-01965 | R | P | MRSP urine | ND | 1(1) | 0.5(0.5) | 0.5(0.5) |
| 37 | 20-02411 | R | P | MRSP ex Gribbles R stifle | ND | 1 | 0.5 | 2 |
| 38 | 20-02894 | R | P | wire implant | ND | 1 | 0.5 | 2 |
| 39 | 20-03055 | R | P | Skin | ND | 1 | 0.5 | 2 |
| 40 | 21-00052 | R | P | pyoderma | ND | 1(1) | 1(1) | 1(1) |
| 41 | 21-02645 | R | P | paw swab | ND | 1 | 0.5 | 2 |
| 42 | 21-02754 | R | P | skin pyoderma | ND | 1 | 0.5 | 1 |
| 43 | 21-02904 | R | P | skin swab | ND | 1 | 0.5 | 2 |
| 44 | 22-00060 | R | P | pyoderma | ND | 1 | 0.5 | 2 |
| 45 | 22-00329 | R | P | wound | ND | 1(1) | 1(1) | 4(4) |
| 46 | 22-00790 | R | P | skin | ND | 1 | 0.5 | 1 |
| 47 | 22-00976 | R | P | joint fluid/ surgical pins | ND | 1 | 0.5 | 1 |
| 48 | TAZ | R | P | P Hill SASH referral paws | ND | 1 | 0.5 | 1 |
| 49 | 22-01920 | R | P | skin | ND | 1 | 0.5 | 1 |
| 50 | V1048056 | R | P | ear | ND | 1 | 0.5 | 1 |
| 51 | V10131194 | R | P | swab | ND | 1(1) | 0.5(0.5) | 1(1) |
| 52 | 22-02274 | R | P | skin | ND | 1 | 0.5 | 1 |
| 53 | V1036957 | R | P | tissue | ND | 1 | 0.5 | 2 |
| 54 | V1037545 | R | P | ear | ND | 1(1) | 1(1) | 1(1) |
| 55 | 24-03318 | R | P | wound swab | ND | 1 | 1 | 1 |
| 56 | 20-01600 | R | P | inguinal area | ND | 1(1) | 1(1) | 1(2) |
| 57 | 20-01965 | R | P | MRSP urine | ND | 1(1) | 1(1) | 1(2) |
| 58 | 20-02411 | R | P | MRSP ex Gribbles R stifle | ND | 1(1) | 1(1) | 1(2) |
| 59 | 20-02894 | R | P | MRSP wire implant | ND | 1 | 1 | 1 |
| 60 | 20-03055 | R | P | MRSP Skin | ND | 1 | 1 | 1 |
| 61 | 21-00052 | R | P | MRSP pyoderma | ND | 1(1) | 1(1) | 1(2) |
| 62 | 21-02645 | R | P | paw swab | ND | 1 | 1 | 2 |
| 63 | 21-02754 | R | P | skin pyoderma | ND | 1 | 0.5 | 1 |
| 64 | 21-02904 | R | P | skin swab | ND | 1 | 1 | 1 |
| 65 | 22-00060 | R | P | pyoderma MRSP | ND | 1(1) | 1(1) | 1(2) |
| 66 | 22-00329 | R | P | wound | ND | 1 | 0.5 | 1 |
| 67 | 22-00790 | R | P | skin | ND | 1 | 0.5 | 2 |
| 68 | 22-00976 | R | P | joint fluid/ surgical pins | ND | 1 | 1 | 2 |
| 69 | TAZ | R | P | paws | ND | 1 | 1 | 1 |
| 70 | 22-01920 | R | P | skin | ND | 1 | 1 | 2 |
| 71 | V1048056 | R | P | ear | ND | 1 | 0.5 | 1 |
| 72 | V10131194 | R | P | swab | ND | 1 | 1 | 1 |
| 73 | 22-02274 | R | P | skin | ND | 1 | 1 | 1 |
| 74 | V1036957 | R | P | tissue | ND | 1 (1) | 0.5 (1) | 1 (2) |
| 75 | N13/4/21 | R | P | surgical site | 64 | 1 | 0.5 | 2 |
| 76 | N13/4/25 | R | P | respiratory | 25 | 1 | 0.5 | 1 |
| 77 | N13/4/52 | R | P | Surgical site | 71 | 1 | 1 | 8 |
| 78 | N13/4/59 | R | P | SST | 498 | 1 | 0.5 | 2 |
| 79 | N13/4/75 | R | P | Surgical site | 316 | 1 (1) | 0.5 (1) | 2 (4) |
| 80 | N13/4/94 | R | P | Surgical site | 496 | 1 | 0.5 | 1 |
| 81 | N13/1/103 | R | P | UTI | 71 | 1 (2) | 0.5 (1) | 2 (4) |
| 82 | N13/4/115 | R | P | Surgical site | 496 | 1 | 0.5 | 2 |
| 83 | N13/4/121 | R | P | Surgical site | 496 | 1 | 0.5 | 2 |
| 84 | N13/4/123 | R | P | SST | 496 | 1 | 0.5 | 2 |
| 85 | N13/1/238 | R | P | ear | 316 | 1 | 0.5 | 2 |
| 86 | N13/1/317 | R | P | ear | 96 | 1 (1) | 0.5 (1) | 2 (4) |
| 87 | N13/1/373 | R | P | ear | 496 | 1 | 0.5 | 2 |
| 88 | N13/1/386 | R | P | Surgical site | 258 | 1 | 1 | 1 |
| 89 | N13/1/421 | R | P | ear | 498 | 1 | 0.5 | 2 |
| 90 | N13/1/438 | R | P | ear | 316 | 1 | 0.5 | 2 |
| 91 | N13/1/446 | R | P | ear | 45 | 1 | 0.5 | 4 |
| 92 | N13/1/480 | R | P | UTI | 71 | 1 (2) | 0.5(0.5) | 2 (4) |
| 93 | N13/1/580 | R | P | STT | 283 | 1 | 0.5 | 1 |
| 94 | N13/1/627 | R | P | SST | 498 | 1 | 1 | 2 |
| 95 | N13/1/649 | R | P | ear | 497 | 1 | 0.5 | 2 |
| 96 | N13/1/651 | R | P | SST | 497 | 1 | 1 | 2 |
| 97 | N13/1/704 | R | P | ear | 496 | 1 (2) | 1 (1) | 2 (4) |
| 98 | N13/1/748 | R | P | SST | 496 | 1 | 1 | 2 |
| 99 | N13/1/839 | R | P | SST | 45 | 1 | 0.5 | 4 |
| 100 | Q13/3/18 | R | P | SST | 316 | 1 (1) | 0.5 (1) | 4 (8) |
| 101 | Q13/3/24 | R | P | ear | 316 | 1 | 0.5 | 4 |
| 102 | Q13/1/35 | R | P | SST | 316 | 1 | 0.5 | 2 |
| 103 | Q13/1/190 | R | P | Other | 496 | 1 (1) | 0.5 (1) | 2 (4) |
| 104 | Q13/1/243 | R | P | SST | 71 | 1 | 1 | 4 |
| 105 | Q13/1/311 | R | P | Surgical site | 71 | 1 | 1 | 4 |
| 106 | Q13/1/317 | R | P | UTI | 316 | 1 (2) | 0.5 (1) | 4 (8) |
| 107 | Q13/1/326 | R | P | surgical site | 71 | 1 | 1 | 4 |
| 108 | V13/6/4 | R | P | SST | 71 | 1 | 1 | 8 |
| 109 | V13/6/5 | R | P | UTI | 71 | 1 | 1 | 2 |
| 110 | V13/6/7 | R | P | SST | 499 | 1 | 1 | 2 |
| 111 | V13/2/18 | R | P | Surgical site | 71 | 1 | 1 | 8 |
| 112 | V13/2/52 | R | P | Surgical site | 71 | 1 | 1 | 8 |
| 113 | V13/2/83 | R | P | ear | 497 | 1 (1) | 0.5 (1) | 2 (4) |
| 114 | V13/2/133 | R | P | SST | 71 | 1 | 1 | 4 |
| 115 | V13/2/140 | R | P | SST | 71 | 1 | 1 | 4 |
| 116 | V13/2/152 | R | P | SST | 71 | 1 (1) | 0.5 (2) | 2 (4) |
| 117 | V13/2/173 | R | P | ear | 497 | 1 | 0.5 | 2 |
| 118 | V13/2/191 | R | P | Respiratory | 71 | 1 | 0.5 | 4 |
| 119 | V13/2/193 | R | P | SST | 84 | 1 | 0.5 | 1 |
| 120 | V13/2/194 | R | P | SST | 500 | 1 (1) | 0.5 (1) | 2 (4) |
| 121 | V13/2/220 | R | P | SST | 497 | 1 | 0.5 | 2 |
| 122 | V13/2/227 | R | P | SST | 501 | 1 | 0.5 | 2 |
| 123 | V13/2/242 | R | P | SST | 497 | 1 (1) | 0.5 (1) | 2 (4) |
| 124 | V13/2/393 | R | P | SST | 71 | 1 | 1 | 4 |
| 125 | V13/2/407 | R | P | SST | 497 | 1 | 0.5 | 2 |
| 126 | V13/2/413 | R | P | STT | 71 | 1 | 1 | 4 |
| 127 | V13/2/440 | R | P | STT | 71 | 1 (1) | 0.5 (1) | 4 (8) |
| 128 | V13/2/441 | R | P | SST | 71 | 1 | 0.5 | 4 |
| 129 | V13/2/475 | R | P | SST | 71 | 1 | 0.5 | 2 |
| 130 | V13/2/488 | R | P | SST | 71 | 1 | 0.5 | 2 |
| 131 | W13/1/4 | R | P | other | 45 | 1 (1) | 0.5 (1) | 4 (8) |
| 132 | W13/1/5 | R | P | SST | 45 | 1 | 0.5 | 4 |
| 133 | W13/1/11 | R | P | ear | 45 | 1 | 0.5 | 4 |
| 134 | W13/1/12 | R | P | SST | 45 | 1 (1) | 1 (1) | 4 (8) |
| 135 | V1186062 | R | P | paw | 64 | 1 | 1 | 1 |
| 136 | V1186211 | R | P | blood | 25 | 1 | 1 | 1 |
| 137 | V1186371 | R | P | groin | 71 | 1 | 1 | 2 |
| 138 | V1188770 | R | P | feet | 498 | 1 | 1 | 2 |
| 139 | V1192734 | R | P | middle ear syringe | 316 | 1 | 1 | 1 |
| 140 | V1194423 | R | P | middle ear syringe | 496 | 1 | 1 | 4 |
| 141 | V1194750 | R | P | ventral abdomen | 71 | 1 | 1 | 1 |
| 142 | V1196147 | R | P | crust wound swab | 496 | 1 | 1 | 2 |
| 143 | V1198070 | R | P | ear | 496 | 1 | 1 | 4 |
| 144 | V1200895 | R | P | tissue | 496 | 1 | 0.5 | 2 |
| 145 | V1200668 | R | P | leg wound | 316 | 1 | 1 | 4 |
| 146 | V1201839 | R | P | tissue | 96 | 1 | 1 | 2 |
| 147 | V1202215 | R | P | groin | 496 | 1 | 1 | 4 |
| 148 | V1202137 | R | P | middle ear | 258 | 1 | 1 | 2 |
| 149 | V1204278 | R | P | hip lesion | 498 | 1 | 1 | 2 |
| 150 | V1204871 | R | P | nose wound | 316 | 1 | 1 | 2 |
| 151 | V1205892 | R | P | tissue | 45 | 1 | 1 | 4 |
| 152 | V1205712 | R | P | tissue | 71 | 1 | 1 | 2 |
| 153 | V1206274 | R | P | skin around eye | 283 | 1 | 1 | 1 |
| 154 | V1206275 | R | P | skin | 498 | 1 | 1 | 1 |
| 155 | V1206277 | R | P | skin | 497 | 1 | 1 | 2 |
| 156 | V1206365 | R | P | ear | 497 | 1 | 1 | 4 |
| 157 | V1206272 | R | P | dorsal crusts | 496 | 1 | 1 | 2 |
| 158 | V1206910 | R | P | Foot swab | 496 | 1 | 1 | 2 |
| 159 | V1207742 | R | P | claw fold | 45 | 1 | 1 | 2 |
| 160 | V1208576 | R | P | NA | 316 | 1 | 1 | 2 |
| **MIC range** | | | | |  | 1 | 0.5-1 | 1-8 |
| **MIC_50_** | | | | |  | 1 | 0.5 | 2 |
| **MIC_90_** | | | | |  | 1 | 1 | 4 |

NA=Not Available; R=Resistance; P=Positive; UTI=Urinary Tract Infection; SST=Skin and Soft Tissue; ND=Not Determined; Van=Vancomycin; Ami=Amikacin; yellow=isolates selected for resistance development.

**Reference**

Nguyen HT, O’ Donovan LA, Venter H, Russell CC, McCluskey A, Page SW, Trott DJ, Ogunniyi AD. 2021. Comparison of two transmission electron microscopy methods to visualize drug-induced alterations of Gram-negative bacterial morphology. Antibiotics (Basel) 10.
